# Toxin-Antitoxin Systems as Multilevel Interaction Systems

**DOI:** 10.3390/toxins6010304

**Published:** 2014-01-10

**Authors:** Nathalie Goeders, Laurence Van Melderen

**Affiliations:** Laboratoire de Génétique et Physiologie Bactérienne, IBMM, Faculté des Sciences, Université Libre de Bruxelles (ULB), 12 rue des Professeurs Jeener et Brachet, Gosselies B-6041, Belgium; E-Mail: ngoeders@ulb.ac.be

**Keywords:** endoribonuclease, repression, trans-activation, proteolysis

## Abstract

Toxin-antitoxin (TA) systems are small genetic modules usually composed of a toxin and an antitoxin counteracting the activity of the toxic protein. These systems are widely spread in bacterial and archaeal genomes. TA systems have been assigned many functions, ranging from persistence to DNA stabilization or protection against mobile genetic elements. They are classified in five types, depending on the nature and mode of action of the antitoxin. In type I and III, antitoxins are RNAs that either inhibit the synthesis of the toxin or sequester it. In type II, IV and V, antitoxins are proteins that either sequester, counterbalance toxin activity or inhibit toxin synthesis. In addition to these interactions between the antitoxin and toxin components (RNA-RNA, protein-protein, RNA-protein), TA systems interact with a variety of cellular factors, e.g., toxins target essential cellular components, antitoxins are degraded by RNAses or ATP-dependent proteases. Hence, TA systems have the capacity to interact with each other at different levels. In this review, we will discuss the different interactions in which TA systems are involved and their implications in TA system functions and evolution.

## 1. Introduction

**Figure 1:**
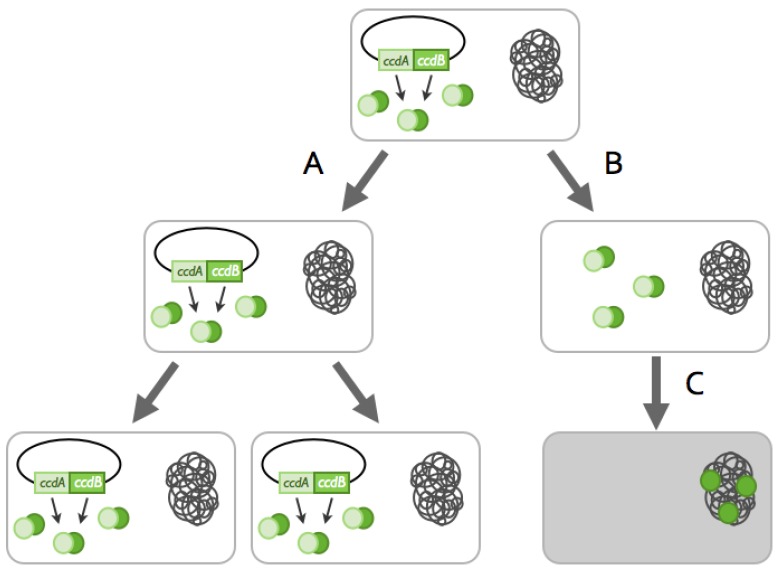
The addiction phenomenon. Toxin-antitoxin (TA) systems participate in plasmid maintenance in growing bacterial populations by a mechanism called addiction or post-segregational killing. Addiction relies on the differential stability of the toxin and antitoxin. **A**: Daughter-bacteria that inherit a plasmid copy encoding the *ccd* (control of cell death) toxin-antitoxin system grow normally. **B**: Daughter-bacteria that do not inherit a plasmid copy still have antitoxin-toxin complexes in their cytoplasm. **C**: The CcdA antitoxin (light green) is degraded by the Lon protease, while the CcdB toxin (dark green) is stable. CcdB is, therefore, liberated from the CcdA-CcdB complex and is able to interact with DNA-gyrase, a class II topoisomerase. The interaction of CcdB with DNA-gyrase inhibits DNA replication and leads eventually to cell death. Addiction leads to the selective killing of plasmid-free daughter bacteria and increases plasmid prevalence in the bacterial population.

Toxin-antitoxin systems (TA) are small modules generally composed of two elements: a stable toxin that targets an essential cellular process and a labile antitoxin that inhibits the toxin’s deleterious activity [1,2,3]. These modules were originally discovered on low copy number plasmids [1,4,5] and coined “addiction” modules (Figure 1). 

Later on, with massive sequencing of bacterial genomes, computer searches led to the discovery of chromosomally-encoded systems [2,6]. Quite surprisingly, TA systems were found to be widespread and abundant in bacterial chromosomes, and this stimulates researcher’s curiosity. So far, up to 88 TA systems were predicted for *Mycobacterium* *tuberculosis*, while some cyanobacteria encode more than 70 predicted toxins and antitoxins. In some Proteobacteria and green sulfur species, up to 2.5% of total open reading frames (ORFs) are predicted to be type II systems [7,8]. Note that the occurrence of type II systems does not depend on the chromosome size. Recent unpublished bioinformatics analyses estimate that a bacterial chromosome and plasmids encode, on average, 3.8 and 0.4 type II systems, respectively [9].

Many research groups became interested in these systems at different levels; toxin activities, gene expression regulation, diversity and, of main interest, the biological roles of these abundant entities [10,11,12,13,14,15,16]. This active field of research led to the identification of different types of TA systems and functions (see below).

### 1.1. Classification of TA Systems

Depending on the nature and mode of action of antitoxins (proteins or RNAs), different types of TA systems have been described [10,17,18,19,20]. Note that toxins are always proteins.

Type I and III systems rely on RNA antitoxins. Type I antitoxins are anti-sense RNAs that bind toxin mRNAs. This leads to translation initiation inhibition and degradation of the RNA duplex (for a recent review, see [20]). Type III antitoxins are composed of repeat motifs that are recognized and bound by the toxins [17,21], leading to their sequestration. 

Type II, IV and V antitoxins are proteins that either sequester, act as antagonists or inhibit the translation of their cognate toxins.

Type II antitoxins are small unstable proteins composed of two domains: an amino-terminal DNA-binding domain (DBD) and a carboxy-terminal region involved in toxin binding [22,23,24]. Note that in some type II antitoxins (e.g., MqsA), the DBD is located in the C-terminal region and the toxin binding domain in the N-terminal part of the protein [25]. Formation of the antitoxin-toxin complex results in toxin sequestration and inactivation. These complexes are also often responsible for negative autoregulation of the TA operon [26,27]. In the case of type IV and V antitoxins, only one example has been described. The type IV CbeA antitoxin acts as the antagonist of its cognate toxin and promotes polymerization of FtsZ and MreB, the toxin targets [19,28]. In type V, the GhoS antitoxin is described as an endoribonuclease that degrades its cognate toxin-encoding mRNA [18]. 

### 1.2. Evolution of TA Systems

Different families of type I TA systems have been described. Some type I loci are found only in a limited number of bacteria, while others are found in different phyla. Up to 26 type I loci have been predicted in the *Escherichia coli* O157: H7 Sakai strain [20]. In contrast to type II and type III systems that spread by horizontal gene transfer, type I loci seem to be inherited vertically and arise by duplication in specific lineages [20]. Although type III systems appear to be prone to horizontal gene transfer, they are less abundant than type II systems [29]. They have been grouped into three families based on toxin sequence similarity, and up to six type III loci have been found in one species [29]. Type III loci are found mainly in Firmicutes and Fusobacteria and to a lesser extend in Proteobacteria, Archaea and on phages [29]. As type III system identification is based on three-dimensional structure similarity so far, further bioinformatics approaches will probably reveal a higher number and diversity of type III systems. As type IV and V systems were discovered recently, their abundance, dissemination and evolution have not been investigated yet.

Type II TA systems are probably the most abundant and the best described class of TA systems. Currently, type II toxins are classified in 12 super-families based on amino acid sequences and three-dimensional structure similarities [7]. Type II antitoxins form 20 super-families and are based on the same criteria [7]. It was thought for some time that each toxin super-family is associated with a specific antitoxin super-family. However, bioinformatics and experimental studies showed that “mixing and matching” occurs, indicating that type II systems have been assembled from these toxin and antitoxin super-families at different occasions by *in situ* displacement, as proposed by Anantharaman and Aravind [7,30,31,32,33,34]. In addition, shuffling between TA types also occurs. The type III ToxN toxin is an endoribonuclease that shares the same fold as the CcdB/MazF type II super-family [21]. Type I toxins are generally small inner membrane proteins that disrupt the proton motive force (PMF), such as the type V GhoT toxin [18,35,36,37]. Strikingly, the type I toxin SymE, which shows endoribonuclease activity, shares the same fold as the MazE type II antitoxin super-family [38], and the type IV CbeA antitoxin presents a RelE fold [39]. Therefore, such evolutionary processes offer a wide range of possibilities for interactions between toxin and antitoxin molecules, as well as with cellular components.

## 2. Multi-Level Interactions between TA Systems

As cited above, TA systems are abundant in bacterial chromosomes [7,40]. Thus, homologous and non-homologous systems co-exist within a bacterial genome (chromosomes and mobile genetic elements (MGEs)) or within the same replicon (either chromosomes, either plasmids). This raises the question of the interaction between homologous and non-homologous systems and how it can impact TA systems’ evolution and activity. 

In this section, we will describe different types of interactions between systems belonging to the same replicon (either on chromosomes or on plasmids) or interactions between chromosomally and MGE-encoded systems. Different levels of interactions can be recognized. Direct interactions between toxin and antitoxin proteins belonging to homologous or non-homologous systems can occur. This can affect TA systems at the level of toxicity or at the level of expression, since in type II systems, antitoxins and/or antitoxin-toxin complexes are able to act as transcriptional repressors. In addition, given that most of type II toxins are endoribonucleases, their activity can impact TA system expression at the post-transcriptional level. We will discuss the implications of these various interactions from an evolutionary perspective. Note that this section will mainly concern type II systems.

### 2.1. TA Systems Sharing the Same Replicon

#### 2.1.1. Cross-Interactions between Non-cognate Antitoxin and Toxin Proteins

Several studies describe analyses of interactions between components of type II systems located in the same chromosome [6,34,41,42]. The case of homologous systems has been well documented, and it appears that interactions between cognate toxins and antitoxins are highly specific despite a good conservation at the amino sequence level. This has been shown notably for the *E. coli mazEF* and *chpB* systems [6] and for 2 *yefM-yoeB* systems of *Staphylococcus equorum* [41], as well as for seven and three RelE/ParE toxins in *Caulobacter crescentus* and *E. coli* O157:H7, respectively [34,42]. The most remarkable example is certainly the case of the 30 functional VapBC system of *Mycobacterium tuberculosis* [8,43,44]. Despite their large number, no cross-interactions were detected between toxins and antitoxins from different systems [8,43].

Cross-interactions between toxins and antitoxins of different systems could result in a strong negative effect. Since a balanced toxin:antitoxin ratio is crucial for survival, perturbation of this ratio by, e.g., the interaction of a given antitoxin with several toxins from different systems could lead to an excess of free toxin and inhibition of cell growth and/or cell death. Proof of principle was obtained since expression of chimera MazF toxins in *E. coli* led to endogenous MazF_K-12_ activation, most likely by competition of these chimera with the endogenous MazF_K-12_ toxin for MazE_K-12_ antitoxin binding [45]. In addition, expression of inactive toxin mutants was used to titrate endogenous toxins and activate the endogenous systems of both chromosomally- and plasmid-encoded TA loci [46,47]. 

Interestingly, cross-interactions between homologous systems might potentially lead to TA system degeneration. We have detected two homologous *relB_-_parE_Sme_* systems on the pSymA megaplasmid of *Sinorhizobium meliloti* [48]. The first system appears to be a *bona fide* TA system, *i.e.*, toxin expression is toxic for *E. coli* and co-expression of the antitoxin restores normal growth. However, in the second system, the predicted antitoxin-encoding gene, *relB_Sme_*_2_, appears to be truncated of its 5’-end, although it shares 93% nucleic acid sequence identity with the 3’ region of *relB_Sme_*_1_ antitoxin from the first system. The predicted toxin-encoding gene *parE_Sme_*_2_ toxin shares 91% nucleic acid sequence identity with the 5’ region of *parE_Sme_*_1_. Cross-talks between these systems were investigated. Surprisingly, ParE2*_Sme_*_2_ is not toxic for *E. coli*, but the predicted RelB2*_Sme_*_2_ retained antitoxin activity against the functional ParE*_Sme_*_1_. Thus, multiple copies of the same system might perturb the subtle balance of TA systems and lead to the decay of one of them. 

**Figure 2:**
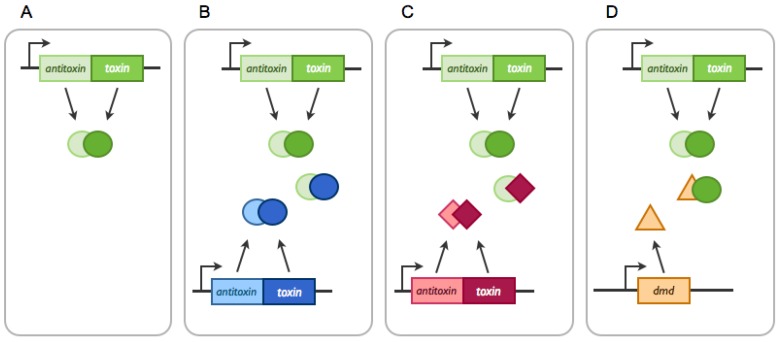
Interactions between TA systems. (**A**) In most cases, antitoxins and toxins only interact with their cognate partners; (**B**) chromosomal systems can interact with homologous systems, such as for anti-addiction; (**C**) TA systems can bind non-canonical antitoxins and toxins, which can lead to network formation and (**D**) a toxin can be inhibited by a protein not related to TA systems, as in the case of Dmd of the T4 phage.

On the other hand, cross-interactions between homologous or non-homologous systems have been described in *M. tuberculosis* (Figure 2) [49,50]. The first network involves direct cross-interactions between homologous *relBE* systems. Non-cognate complexes of toxins and antitoxins are able to bind promoter regions, suggesting possible expression cross-regulation between these systems [49]. The second network includes direct cross-interactions between non-homologous *mazE-vapC* and *mazEF* systems [50]. Non-cognate toxin-antitoxin interactions were detected upon overexpression in *E. coli*. It was proposed that such well-regulated cross-interactions between homologous or non-homologous systems could evolve into networks acting cooperatively to facilitate *M. tuberculosis*’ adaptation to its environment during infections [49,50,51]. However, further experiments are needed to assess the *in vivo* relevance of these potential networks, since deletion of the *relE* genes did not affect *M. tuberculosis* growth in macrophages nor growth or survival in an *in vivo* murine model [52]. These complex interactions might be specific to bacterial species/isolates carrying a high number of TA systems, such as *M. tuberculosis*.

Thus, in most cases, TA systems co-existing on the same replicon appear to not “see” each other. Given the deleterious consequences of accidental toxin activation, the absence of cross-talk might have been selected by evolution. However, some TA systems seem to be involved in complex networks involving multiple systems. The prevalence and the functionality of these networks remain to be shown, but they could have appeared with fine-tuned adaptation responses to environmental conditions.

#### 2.1.2. TA systems Trans-Activation by Non-cognate Toxins

As most type II toxins are endoribonucleases, the expression of these toxins may impact gene expression in general (see below) and, in particular, that of TA systems. It was shown that overexpression of *E. coli* toxins (RelE, MazF, MqsR, HicA and HipA) trans-activates the *relBE* system by alleviation of transcriptional repression [53]. In addition, post-transcriptional regulations of newly synthesized mRNAs occur, as toxin overexpression might lead to the degradation of the antitoxin-encoding portion of mRNAs and accumulation of the toxin-encoding region [53]. As a result, this could generate an unbalanced antitoxin:toxin ratio, leading to toxin accumulation. However, this might not be a universal phenomenon, as the antitoxin transcript of a plasmidic system was recently shown to be unaffected by its cognate toxin. The authors proposed that this would allow for reversible activation of TA systems [54]. Recently, the Wood group showed that the *E. coli* *mqsRA* system activates the type V *ghoST* system [55]. The molecular mechanism relies on the endoribonuclease activity of the type II MqsR toxin. MqsR activates the expression of *ghoT* by cleaving the antitoxin-encoding portion of the *ghoS-ghoT* messenger [55]. 

Trans-activation of *E. coli* TA systems has also been reported upon overexpression of phage and plasmid-encoded toxins, e.g., overexpression of the type II Doc toxin encoded by the P1 phage leads to *relBE* trans-activation [56] and that of the *Shigella* plasmid, pMYSH6000 VapC, activates the type II *yefM-yoeB* system [57]. However, it is not known whether these toxins specifically degrade the antitoxin-encoding portion of the messenger. In the same line of reasoning, one could also imagine that in the addiction phenomenon, plasmid-encoded toxins induce a chain reaction by indirectly activating chromosomally-encoded toxins. 

In general, the molecular mechanisms underlying trans-activation remain to be determined. In the case of type II systems, it is not clear how the first step of trans-activation occurs. How is the non-cognate toxin able to alleviate repression of a given TA operon? In the case of cognate systems, it has been shown by several groups [26,27,58] that disruption of the antitoxin:toxin ratio in favor of toxin allows the formation of a specific toxin-antitoxin complex unable to mediate repression, thereby allowing re-synthesis of both components. However, in the case of a non-cognate toxin, one would have to imagine that non-cognate complexes are formed and share the same properties. Another hypothesis relies on the fact that all these experiments have been performed with toxins showing translation inhibition activity. Overexpression of these toxins will lead to translation arrest. As antitoxins are unstable, they would be rapidly degraded, leading to repression alleviation. Therefore, trans-activation could be a secondary effect of translation inhibition. However, as some specificity is observed (a given toxin does not trans-activate all the TA systems), the mechanisms involved might be subtler.

### 2.2. TA Systems Belonging to Different Replicons

Interactions between TA systems located on different replicons might be viewed as competition between the different systems and, as a consequence, between replicon. This can be viewed as an “arms-race” between TA systems from different locations that drives the evolution of TA systems. 

#### 2.2.1. Arms-Race between Chromosomally- and Plasmid-Encoded TA Systems: The Anti-addiction Model and Beyond

As explained in Figure 1, addiction relies on the capacity of a plasmid-encoded TA systems to inhibit cell growth and/or kill daughter cells devoid of a plasmid copy. “Protection” against this can be achieved if a chromosomally-encoded antitoxin is able to neutralize the plasmid-encoded toxin (Figure 2) [59,60]. On the one hand, this “anti-addiction” phenomenon provides selective advantage to the chromosomally-encoded system by allowing the survival of plasmid-free daughter cells. On the other hand, the selective pressure then lies on the plasmidic toxin. Toxins that are not recognized by the chromosomally-encoded antitoxins would be selected, as they increase the plasmid prevalence in the population. Few modifications appear to be required to modify antitoxin specificity. The group of Kedzierska showed that one specific mutation in the plasmid-encoded Txe toxin allows for recognition by both its cognate antitoxin and a chromosomally-encoded antitoxin [61]. Anti-addiction might thus serve as a driving force for plasmid-encoded toxins evolution. 

Plasmid-encoded TA systems in which toxins are able to evade chromosomally-encoded antitoxins have been identified, such as the F plasmid *ccd_F_* system and the chromosomally-encoded *ccd_0157_* system in *E. coli* O157:H7 [62]; and the R100 plasmid *par* locus and the chromosomally-encoded *mazEF* and *chpB* systems [63,64]. In addition, molecular evolution analyses revealed that CcdB-like toxins encoded by plasmids are under strong negative selection, indicating that these sequences are very constrained, while chromosomally-encoded toxins are under neutral selection, indicating that these sequences are devoid of functions [65]. 

#### 2.2.2. Arms-Race between Chromosomally-, Plasmid- and Phage-Encoded TA Systems: The Phage Defense Model, RM, Abi and Beyond

TA systems were proposed to be bacterial defenses against phages. Defense systems either use self-/non-self-discrimination systems, like restriction-modification (RM) systems and DNA phosphorothioation (DND) systems, or trigger cell death of infected cells [66]. As recently underlined by the Kobayashi group, RM systems share many features with TA systems [67]. On the other hand, Abi (abortive infection) systems hinder the propagation of phages in bacterial populations by inducing the death of infected cells. It appears that some TA systems, like type I *hok*/*sok*, type II *rnlAB* and type III *toxIN* systems, function as Abi systems [17,68,69]. As both ToxI and Sok are RNAs, the alteration of host translation following phage infections probably affects the antitoxin:toxin ratio and leads to toxin activation. In both cases, phage maturation is slowed down and burst size reduced [17,68]. Note that both *hok/sok* and *toxIN* are encoded on plasmids [17,68]. In the case of the *E. coli* *rnlAB* system*,* the labile RnlB antitoxin is also degraded during phage infection as host protein expression is arrested. Abi occurs upon infection by a *dmd* T4 mutant (see below). In this case, the RnlA toxin, an endoribonuclease [70], is activated and degrades phage late mRNAs, thus aborting infection [69]. 

However, as happens in the perpetual arms race between bacteria and phages, phages have found remedies to inhibit toxin activities. Phages have evolved different mechanisms to avoid this, e.g., antitoxin mimicry [71,72], protease inhibition [73] or the hijacking of TA systems [72]. The T4 phage encodes Dmd, a protein functionally equivalent to RnlB and able to inhibit RnlA endoribonuclease activity (Figure 2). As RnlA can no longer degrade T4 mRNAs, T4 infection is effective. In addition, Dmd was shown to counteract the LsoA toxin, which belongs to the homologous *lsoAB* system in *E. coli* O157:H7 [71]. Thus, T4 encodes an antitoxin that has evolved to recognize multiple toxins in order to escape Abi systems. Phage escape to the Abi *toxIN* system is mediated by the expansion of the *toxI* repeat motif that sequesters the ToxN toxin or by the acquisition of a complete *toxIN* copy by recombination [72]. Other phages have evolved different mechanisms to escape TA/Abi systems. The T7 phage has developed a general response against type II TA/Abi systems. Instead of inhibiting toxin activities, T7 protects antitoxins from degradation by expressing the Gp4.5 protein, which inhibits the ATP-dependent Lon protease [73].

Interestingly, TA systems involved in the arms race with MGEs are located themselves on MGEs. The *rnlAB* and *hok/sok* systems are located on prophages (CP4-57 and Qin prophages) in *E. coli* K-12. The *lsoAB* is on a cryptic plasmid of *E. coli* O157:H7. This again points towards the MGEs origin of TA systems and might simply reflect competition between selfish entities at the level of TA systems themselves or MGEs. 

## 3. Interactions of Antitoxins and Toxins with Cellular Components

### 3.1. Interactions Involving Toxins

#### 3.1.1. Interfering with Translation

Blocking translation appears to be the “favorite” inhibitory mechanism of type II toxins [74,75,76,77,78,79]. The most common mechanism relies on mRNAs cleavage in a translation-dependent or -independent manner.

Interactions with the ribosome: Most of the toxins belonging to the type II RelE toxin family cleave mRNAs in a translation-dependent manner by entering the ribosomal A site. These toxins usually cleave mRNAs between the second and the third nucleotide of codons or between codons [31,80,81]. Some RelE-like toxins appear to be devoid of specificity [31,74,80], while others, like *E. coli* YoeB_K-12_, YafQ_K-12_ and HigB from *Proteus vulgaris*, are highly specific [81,82,83,84]. YoeB_K-12_ cleaves predominantly between the start and the second codon, while YafQ_K-12_ and HigB cleave at the AAA lysine codons [81,82,84]. The *E. coli* RatA, which represents another family of toxins, inhibits translation at the initiation step by specific association with the 50S sub-unit and, thereby, inhibiting 70S ribosome formation [85].

Direct interactions with RNA targets: Interestingly, some toxins from the RelE family cleave mRNAs in a translation-independent manner like toxins from the MazE family (see below). The *E. coli* MqsR toxin is an endoribonuclease that cleaves GC[U/A] motifs [74,86]. RelE-like toxins from *Brucella abortus* and *Helicobacter pylori* also act in a translation-independent manner *in vitro* [87,88]. Type II toxins from the MazF family are endoribonucleases that cleave mRNAs specifically at either three-, four-, five- or seven-base recognition sequences [54,75,77,89,90]. Some MazF-like toxins have specialized to target specific transcripts. MazF_Sa_ and the plasmidic PemK_Sa_ toxins from *Staphylococcus aureus* recognize UACAU pentad and UAUU tetrad sequences, respectively [54,75]. Cleavage of these motifs affects virulence in opposite ways, since the UACAU pentad is abundant in transcripts encoding virulence factors (e.g., SraP adhesive factor), while the UAUU tetrad is underrepresented in these transcripts [54]. Thus, MazF_Sa_ down-regulates virulence gene expression [75]. The MazF_-mt3_ and MazF_-mt7_ of *M. tuberculosis* cleave pentad sequences. As for PemK_Sa_, these motifs are underrepresented in specific genes involved in virulence [77]. Thus, these toxins appear to be involved in virulence gene expression control. Likewise, a plasmid-encoded member of the MazF family, the Kid toxin of plasmid R1, cleaves the intercistronic region of the *copB-repA* mRNA, thereby positively regulating the R1 copy number [91]. 

Recently, the three-dimensional structure of the RnlA type II toxin was solved [92]. RnlA represents a novel type II toxin family, as it does not share the fold of known toxin families. LsoA, a second member of this new family, shares 45% similarity at the amino acid sequence level with RnlA [71]. Both toxins show non-specific endoribonuclease activity [70,71]. Finally, members of the VapC toxin family in *Shigella flexneri* and *Salmonella enterica* show endoribonuclease activity against initiator tRNAs [93]. 

Direct interactions with factors involved in translation: Some enzymes involved in translation constitute targets for type II toxins. The P1 phage Doc toxin is a kinase and phosphorylates the EF-Tu elongation factor on a conserved threonine residue. Phosphorylated EF-Tu is unable to bind aminoacylated tRNAs, and this leads to translation inhibition [94]. The *E. coli* HipA toxin is also a kinase [95,96]. It phosphorylates the glutamyl-tRNA synthetase on serine 239, thereby inactivating it. This, in turn, leads to accumulation of uncharged tRNA^Glu^ and translation inhibition [96]. Note that it was previously thought that HipA was targeting EF-Tu [95].

#### 3.1.2. Interfering with DNA Replication

Direct interactions with DNA-gyrase: Plasmid-encoded CcdB_F_ and ParE_RK2_ type II toxins target DNA-gyrase, a type II topoisomerase [97,98,99,100]. CcdB_F_-resistant mutants were isolated and mapped in the *gyrA* gene encoding the GyrA sub-unit (mutation GyrA_462_) [101]. CcdB_F_ interacts with gyrase in an open conformation and stabilizes the DNA-gyrase complexes. This results in inhibition of the DNA religation step and leads to DNA double-strand breaks formation, inhibition of replication, SOS induction, cell filamentation and, eventually, cell death [4,101,102]. ParE_RK2_ toxin was shown to inhibit DNA-gyrase and induce double-strand break formation *in vitro* [99]. ParE_2_ from *Vibrio cholerae* was also shown to interact with the GyrA sub-unit, although to distinct sites from CcdB_F_, and unlike CcdB_F_, requires ATP to stabilize DNA-gyrase cleavable complexes. This indicates that ParE inhibits DNA-gyrase in a different manner than CcdB_F_ [100]. Another member of the ParE family (ParE2 from *E. coli* 0157:H7) was shown to induce the SOS response and to colocalize with the nucleoid [34].

#### 3.1.3. Interfering with Peptidoglycan Synthesis

The type II Zeta-like toxin from *Streptococcus pneumoniae* (PezT) is a kinase that inhibits cell wall synthesis. PezT phosphorylates uridine diphosphate-N-acetylglucosamine (UNAG), a peptidoglycan precursor [103]. Phosphorylated UNAG inhibits MurA, which catalyzes the first step of peptidoglycan biosynthesis. Therefore, the expression of this toxin leads to cell lysis, especially of fast-growing cells [103].

#### 3.1.4. Interfering with Inner Membrane

Type I toxins are small hydrophobic proteins, with the exception of SymE (see above). They are predicted to contain an α-helical transmembrane domain [35,36,37]. Some of them are likely to possess a cytoplasmic or periplasmic domain. These toxins are toxic at a high level, and for some of them, they make pores and disrupt the membrane potential, leading to the “ghost” phenotype and, eventually, cell lysis [104]. Some of these toxins have physiological effects in addition to membrane damage. For example, *E. coli* LdrD leads to nucleoid condensation and affects gene expression, notably that of SoxS, the regulator of the superoxide stress response [104]. 

#### 3.1.5. Interfering with Cell Division

The *cbeA-cbtA* system is the only representative of type IV TA systems [19]. The CbtA toxin inhibits the polymerization of MreB and FtsZ in *E. coli*, two essential proteins involved in the cytoskeleton and cell division, respectively [28]. *In vitro*, CbtA inhibits ATP-dependent polymerization of MreB and GTP-dependent polymerization of FtsZ. Interestingly, the two functions of CbtA can be split: the amino-terminus is responsible for FtsZ interaction, while the carboxy-terminus is for MreB interaction. To our knowledge, this is the first example of a toxin belonging to a TA system that interacts with two distinct targets.

### 3.2. Interactions Involving Antitoxins

Canonical type II antitoxins are capable of binding DNA via their DNA-binding domain (DBD) [22,23,24]. Some antitoxins are devoid of a DBD, such as the *E. coli* O157:H7 Paa1 and Paa2 antitoxins [34] and the *Streptococcus pyogenes* epsilon antitoxin [105,106]. These types of antitoxins are part of type II-specific systems that are composed of a third component encoding a transcriptional regulator.

#### 3.2.1. Direct Interactions with TA Promoters

Antitoxins and/or antitoxin-toxin complexes have the capacity to mediate transcriptional autoregulation. Antitoxins and/or antitoxin-toxin complexes bind in general to palindromic sequences located in the promoter region. These repressors sense the level of antitoxin and toxin proteins. An excess of toxin destabilizes the repressor complex and leads to derepression to adjust a steady-state level of both proteins. This property is called conditional cooperativity [26,27,58]. Mathematical models show that conditional cooperativity and stochastic increase of toxin levels can lead to a switch between the growing and non-growing state and can induce persister cell formation [107,108]. Further layers of regulation can be involved, as in the case of the plasmidic Axe-Txe system of *Enterococcus faecium*. This system contains two promoters. The major one, located upstream of the antitoxin gene, is cooperatively regulated by the antitoxin-toxin complex. A second promoter, which is embedded in the antitoxin CDS, could be involved in antitoxin:toxin ratio regulation in cooperation with the modulation of toxin transcript stability [109]. Furthermore, conditional cooperativity is not universal. Indeed, in the case of the *mqsRA* system, the toxin destabilizes the MqsA-DNA complex. This is due to a partial overlap (Arg61) of the MqsA DBD and the toxin binding site [110]. 

Despite antitoxin diversity, a limited number of DNA-binding domains are found in antitoxins, *i.e.*, ribbon-helix-helix fold (CcdA, ParD), helix-turn-helix (MqsA, RelB) and SpoVT/AbrB-type DNA-binding domains (VapB, MazE) [25,111,112,113,114,115]. Antitoxins bind specific palindromes that can be inverted repeats, alternating palindromes or long and short adjacent palindromes [25,115,116,117].

#### 3.2.2. Direct Interactions with Non-TA Promoters

The *E. coli* MqsA and HipB antitoxins have the capacity to regulate the expression of specific genes in addition to their own operon. The Wood group recently showed that MqsA, as well as MqsA-MqsR complexes negatively regulate the expression of *rpoS*, the general stress response sigma factor [25,118]. They also show that under oxidative stress, MqsA is degraded by the ATP-dependent Lon protease, leading to derepression of *rpoS* by MqsA [118]. Amongst the genes regulated by sigmaS is the master regulator of mobility FlhDC. CsgD, the master regulator of biofilm formation, is also regulated by MqsA [119]. Furthermore, MqsA also regulates expression of other genes involved in biofilms, the folding of periplasmic proteins and the inhibition of replication (*mcbR*, *spy*, *cspD*) [25,120]. Hence, MqsA seems to be a key transcriptional regulator of stress response and biofilm formation. A further layer of regulation comes from the type II DinJ antitoxin, which also regulates RpoS. In contrast to MqsA, DinJ acts indirectly by repressing *cspE,* a positive regulator of RpoS [121]. 

A bioinformatics search for the palindrome bound by the HipB antitoxin identified a sequence upstream of 33 genes of diverse functions, *i.e.*, persistence, metabolism, transcriptional regulation and mismatch repair. Experimental validation was obtained for the *relA*, *eutH* and *fadH* promoter regions [117]. RelA is involved in (p)ppGpp synthesis and required for the high persistence phenotype of the *hipA7* mutant [122]. *eutH* and *fadH* encode an ethanolamine transporter and a 2,4-dienoyl-CoA reductase, respectively. 

#### 3.2.3. Direct Interactions with Chaperones and ATP-Proteases

Type II antitoxins are degraded by ATP-dependent proteases. Antitoxin instability is likely due to the fact that most have unstructured N-terminal parts and fold only upon toxin binding [123]. While most antitoxins are a substrate for Lon protease, some are degraded by ClpXP (Phd, PaaA) and ClpAP (MazE). Most antitoxins have half-lives of ~15–20 min, while toxins are stable. Note that the SymE type I toxin is degraded by Lon [38]. Conditional degradation has been shown for MqsA. This antitoxin is stable for up to 60 min in steady-state conditions. Upon oxidative stress, MqsA is very unstable, and its half-life is estimated at around 1.5 min [118]. Recently, the group of Gerdes showed that Lon-dependent degradation of the RelB and YefM antitoxins relies on Poly-P (poly-phosphate) [124]. RelB and YefM are stable in a *ppk-ppx* mutant, unable to synthesize Poly-P. 

Recently, a novel type of type II TA system was identified in *M. tuberculosis*. This system has been quoted as TAC (toxin-antitoxin-chaperone) [125]. This system is composed of a HigA antitoxin, a HigB toxin and a SecB-like chaperone. In the absence of the chaperone, HigA aggregates and is rapidly degraded; thus, the chaperone is essential for antitoxin activity [125].

#### 3.2.4. Direct Interactions with RNAses

Type I RNA antitoxins interact with the transcripts encoding their cognate toxins. Some antitoxins, like IstR, SymR and Sok, bind in the 5’ untranslated region (5’UTR) to prevent translation of the toxin, while others, like SR4, are complementary to the 3’ region of the toxin mRNA [104,126]. In both cases, the toxin-antitoxin RNA duplexes are degraded by RNAses. For instance, the Hok/Sok duplex is degraded by RNAse III and Sok by RNase E [127,128].

In contrast to other species, the RNAse III of *Bacillus subtilis* is essential. Interestingly, this is due to the presence of two type I systems located on the Skin and SPß prophages [129]. In the absence of the two prophages, the deletion of RNAse III-encoding gene *rnc* is viable and does not affect the growth rate. Durand *et al.* showed that RNase III is responsible for the degradation of the *txpA* and *yonT* toxin transcripts when paired with their respective antitoxins.

## 4. Conclusions

In addition to antitoxin-toxin cognate interactions (*i.e.*, RNA-RNA, RNA-protein, protein-protein), interactions between TA systems occur at different levels (transcriptional and post-transcriptional levels). Even if these interactions occur accidentally, they probably shape the evolution of TA systems. The case for TA systems involved in the arms race between chromosomally-encoded loci and those located on MGEs is notable. TA systems might also form complex networks possibly involved in bacterial adaptation. In addition to these “intra” and “inter” TA loci interactions, both components of TA systems interact with cellular components [DNA (e.g., autoregulation), RNAs (e.g., endoribonucleases) and proteins (e.g., DNA-gyrase)] and at multiple levels. Toxins interact with their targets, which become more diverse as more toxins are characterized. Some of these toxin-target interactions reflect the adaptation of particular systems to their location; for example, the plasmid-encoded MazF/Kid/PemK toxin that regulates the plasmid copy-number or the chromosomally-encoded MazF toxins that modulate the amount of specific transcripts. Besides, specific antitoxins regulate the expression of specific genes at the transcriptional level. These small unstable proteins are degraded by ATP-dependent proteases, and some of them need the help of a SecB-like chaperon to fold properly and be active.

Thus, these simple modules have evolved multiple interactions between each other and with their host bacteria that contribute most likely to their evolutionary success. 
